# A Literature Review of the Quality of Life, Health Status and Prosthesis Satisfaction in Older Patients With A Trans-tibial Amputation

**DOI:** 10.33137/cpoj.v3i1.33640

**Published:** 2020-05-21

**Authors:** S Brunelli, C Bonanni, C Foti, M Traballesi

**Affiliations:** 1 Fondazione Santa Lucia, Scientific Institute for Research, Hospitalization and Health Care, Rome, Italy.; 2 Physical and Rehabilitation Medicine, Tor Vergata University of Rome, Rome, Italy.

**Keywords:** Quality of Life, Amputation, Satisfaction, Rehabilitation, Review, Prosthesis, Amputee, Lower Limb Amputation, Health Status

## Abstract

**BACKGROUND:**

Several reviews have been published regarding quality of life (QoL) and Health Status (HS) in persons with lower limb amputation (LLA). However, little has been discussed in the literature with respect to older populations (i.e. age>60 years) with trans-tibial amputation. Furthermore, the perceived satisfaction with prosthesis is another important aspect for consideration in the amputees’ life.

**OBJECTIVE:**

The purpose of this review was to evaluate the impact of trans-tibial amputation on the QoL, HS and prosthesis satisfaction, in order to determine the appropriate intervention to improve these aspects in older population of trans-tibial amputees (TTA).

**METHODS:**

Research articles, published between January 2000 to March 2019, were found using Scopus, PubMed and Google Scholar databases. The methodological quality of the selected articles was assessed using the Critical Review Form-Quantitative Studies checklist.

**RESULTS:**

Ten articles that met the inclusion criteria were selected. In these papers, we can summarize that people with trans-tibial amputation have a better QoL compared to those with above knee amputation. Moreover, physical functioning and mobility are the most influencing factors for QoL and HS in older people with lower limb amputation. Finally, the prosthesis weight reduction may improve satisfaction with the prosthetic limb.

**CONCLUSION:**

Efforts have to be made in order to improve mobility in older population with transtibial amputation for better QoL and HS. This can be accomplished by means of adequate rehabilitation, pain management and an accurate choice of appropriate prosthetic components. We observed that the quality of evidence in the literature available is inadequate and future research would benefit from more prospective observational cohort studies with appropriate inclusion criteria and larger sample sizes to better understand the QoL and HS in this population.

## INTRODUCTION

The lower limb amputation is a dramatic event that can negatively impact functional mobility, perceived health status (HS) and quality of life (QoL) of a person.^1^ In the past, QoL and HS outcome have not been considered as an important goal for a rehabilitation project. Moreover, QoL and HS assessments are rarely performed in routine clinical practice and in clinical trials, particularly in the field of prosthetics. The hypothesis that a better functional outcome (i.e. mobility and performance with the prosthesis), is associated with improved QoL is not always confirmed, as patients’ perception of overall well-being and satisfaction could be different from the predictions of physicians.^2^ For a complete and accurate assessment of multiple aspects of a person’s status, it is important to differentiate between HS and QoL.^3^ Quality of life has been defined by the World Health Organization (WHO) as *“individuals’ perceptions of their position in life in the context of the culture and value systems in which they live and in relation to their goals, expectations, standards and concerns*”.^4,5^ Health Status is often indistinct from QoL. The assessment of perceived Health Status has the goal of evaluating a persons’ perception of his or her disease influenced by the complex interactions of social, emotional and physical functioning.^6^

The evaluation of QoL and HS, by means of reliable questionnaires, might determine which are the most influencing factors and thereby helping the rehabilitation team or the healthcare services to improve care of persons with limb amputation.^7^ A proper investigation of QoL in people with amputation could rely on the use of specific instruments developed for this purpose, such as: Trinity Amputation and Prosthesis Experience Scales (TAPES)^8^ that investigate these domains: psychosocial adjustment; social, functional and athletic restriction; prosthesis satisfaction; pain and other medical problems, or the Prosthesis Evaluation Questionnaire (PEQ)^9^ which investigates ambulation, appearance, frustration, perceived response, residual limb health, social burden, sounds, utility and well-being.

A comfortable prosthetic device allows amputees to walk and carry out daily activities without pain and could increases their satisfaction, independence and activity level.^10^ Even an aesthetically acceptable prosthetic device might favourably influence the social reintegration of the patient. A comprehensive life assessment of people with lower limb amputation must take into account their satisfaction with the prosthesis. Those satisfaction aspects are included in some items of TAPES and PEQ. Furthermore, the SAT-PRO (satisfaction with prosthesis) was developed specifically for this purpose.^11^

Many studies have investigated functional outcome, functional status, mobility level and the predictor factors in LLA.^12,13^ However, rarely those data are associated with QoL, HS or satisfaction with the prosthesis. Moreover, studies rarely focus on different populations of LLA, in a way that the results can be differentiate between elderly or younger people with trans-tibial or trans-femoral amputation.^14^

It should be noted that poor QoL in a person with lower limb amputation may depend not only on physical disability but also on pain, in particular low back pain or artrithis^15,16^ or phantom pain.^17^ In addition, traumatic amputation at young age is associated with better QoL.^18^ Some reviews about QoL and HS are available, however they are related exclusively to a general sample of LLA.^14,19,20^

This literature review was undertaken with a purpose to support or refute any or all of the following assumptions: a) TTA have better mobility capacity than TFA^21^; b) people above 65 years old present lower physical performance than younger patients; and c) the performance status of older patients after amputation is generally poor.^22^ With these observations in mind, the aim of this review was to analyse the QoL, HS and satisfaction with the prosthesis in a specific group of trans-tibial amputees (i.e. age>60y).

## METHODOLOGY

### Search strategy

Two authors, SB and CB, independently conducted a search in the spring 2019 to find related research articles using Scopus, PubMed and Google Scholar databases. The electronic literature search included articles published from January 2000 to March 2019, using the keywords “amputee”, “lower limb amputation”, “trans-tibial”, “below-knee”, “health status,” “quality of life”, “outcome” and “satisfaction”. We have included the keyword “outcome” as sometimes the keyword of a study was the functional outcome and the QoL or HS described only as secondary aims. Moreover, “SF-36”, “WHO QOL-BREF”, “PEQ”, “PPA (Prosthetic Profile for Amputee^23^)” and “TAPES-R”, have also been searched as these are the most used tools for the measurements of QoL or HS in LLA. Combinations of keywords were made in order to refine the search results by using Boolean terms ‘AND’ and ‘OR’.

### Review process

The reference lists of all screened articles were also examined for any potentially eligible studies. Reviews, case reports, congresses abstracts, comments, editorials, guidelines, letters and studies not in English were excluded. Articles that focused on individuals with upper limb amputation or solely on individuals with above knee amputation or on people with mean age<60 were excluded. The authors performed a second screening by reading the full-text of the selected articles, to understand if they could obtain data on QoL, HS or satisfaction with the prosthesis based on the following inclusion criteria: 1) persons diagnosed with TTA; 2) studies investigating QoL and/or HS and/or satisfaction with the prosthesis in persons aged >60 years; 3) use of standardized evaluation measures. As all data was drawn from literature and as such no informed consent or ethical approval was needed for this study.

### Study quality

The methodological quality of the selected articles was assessed using the Critical Review Form-Quantitative Studies checklist.^24^ The checklist consists of 15 questions pertaining to the quality of reporting, internal validity, external validity, and power of the studies. Higher scores representing better quality. Most questions were answered as “1” for a yes or “0” for a no. Some questions had the option “unable to determine”, these questions were excluded from the checklist.^24^

## RESULTS

### Article selection

**Figure 1:**
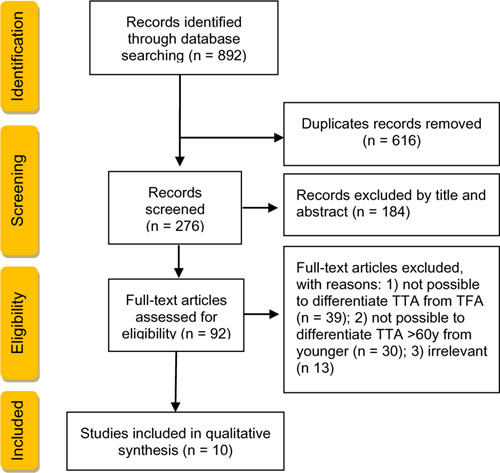
Flowchart summarizing the study selection process.

An initial electronic database search obtained 892 articles. An identification of duplicates excluded 616 articles. Screening of the title and abstract further excluded 184 articles because they were not investigating the impact of a trans-tibial amputation on the QoL, HS or satisfaction with the prosthetic limb. Eighty-two articles were removed after reviewing the full texts. In total, 10 articles were selected for the purpose of this literature review (Figure 1).

In our review of literature, no randomized controlled trial studies were found on the topic of interest. The majority were observational studies (n=5) and cross-sectional (n=3) studies while the others were cross-over (n=1) or retrospective studies (n=1). The results of the four studies,^2, 25–27^ which evaluated QoL in older TTA are summarized in Table 1. Four studies^17,28–30^ evaluated HS in this population and their findings were summarized in Table 2. Moreover, Table 3 shows two studies on patient level of satisfaction with the prosthesis.^31–32^ The only study that investigated both HS and QoL,^27^ is included in Table 1, as the main results were related to QoL.

**Table 1:** Overview of studies reporting QoL data.

Authors	Evaluation tools	Study protocol	Characteristics of patients	Aim of study	Results	Critical review form-total items
Harness et al. (2001)^2^	PEQ	Follow up	60 dysvascular TTA (mean age 65.9 ± 1.4 years) with successful use of current prosthesis for a minimum of 6 months	Determining QoL of a population of TTA who were successful prosthetic users	The response to the PEQ domains of perceived responses, frustration, social burden, overall well-being and overall satisfaction were above 65% of the midline of the PEQ scores. The domains “ambulation” and “transfer” showed less favorable responses. Statistical study of the relationships between domains showed these correlations: a. “residual limb health” and “prosthetic appearance” with “social burden” “satisfaction” b. less “pain” with “satisfaction”. c. “ability to ambulate” with “satisfaction” d. “transfer ability” with “satisfaction” and with decreased “social burden” e. “pain” and “residual limb health” with “ability to ambulate” f. “social burden” with “ambulation”	10/12*
Norvell et al.(2011)^25^	SWLS	Prospective cohort study	87 LLA (8 TFA, 52 TTA, and 27 Transmetatarsal amputees). TTA mean age was 61.5±9.1 years. Only 43 individuals reached 12-month follow-up	Examining the association of “mobility success” with satisfaction with mobility and satisfaction with life; comparing rates of mobility success between various amputation levels; evaluating factors associated with mobility success	This study did not find a significant difference in mobility results between TFA and TTA. This could depend on the very small number of TFA. 50% of TTA were satisfied with their mobility. No differences were found between TTA and transmetatarsal amputees in terms of mobility satisfaction. The satisfaction with life was 28% higher in amputees with higher mobility score. There is also a correlation between higher mobility score and satisfaction with mobility	14/15
Cox et al. (2011)^26^	WHO QOL-BREF	Observational study	87 LLA (64 TTA, 23 TFA) Mean age: 62±9.9 years. 35 males and 52 females. All TTA males were > 60 years. 78% of TTA females were >60 years	Determining the QoL of diabetic LLA and the relationship with gender, age and amputation level	TTA showed a better QoL. Females were found to have higher scores in the QoL domains (physical health, physiological, social relationship and environment) than males, even if 40% had a transfemoral amputation. This might depend on the younger age of the females. Females across the age groups had a significantly higher QoL average scores than males	14/15
Quigley et al.(2016)^27^	TAPES-R and modified version of SF-36 (v2)^33^	Cross-sectional study	33 LLA (23 TTA (mean age 68±10 years), 10 partial foot amputees (63 ± 10 years)	Comparing QoL in people with partial foot amputation secondary to peripheral vascular disease and determining factors influencing QoL	The statistic analysis showed no significant differences in the SF-36v2 between TTA and partial foot amputation. Age was the only variable, which concurred significantly with QoL, while level of amputation did not	14/15

**Abbreviations**: LLA, lower limb amputees; TTA, transtibial amputees; TFA, transfemoral amputees; WHO QOL-BREF, World Health Organization Quality of Life Scale; QoL, quality of life;; PEQ, Prosthesis Evaluation Questionnaire; SF-36, Short-Form General Health Survey; SWLS, Satisfaction with Life Scale; TAPES-R, Trinity Amputation and Prosthesis Experience Scale-Revised.

* Some questions had the option “unable to determine”. These questions were excluded from the checklist and this was the reason why some of selected studies might have a maximum score of less than 15.

**Table 2:** Overview of studies reporting HS data.

Authors	Evaluation tools	Study protocol	Characteristics of patients	Aim of study	Results	Critical Review Form-TOTAL items
Van der Schans et al.(2002)^17^	RAND-36	Cross-sectional study	437 LLA, 62% TTA. 71% males. Mean age 65±15 years (8% of the sample was older than 75 years)	Describing health-related quality of life in LLA and investigating potential determinants: including phantom pain age, sex, level of amputation, amputation reason, phantom or stump pain and walking distance	Health-related quality of life was positively influenced by a) absence of phantom pain, b) walking distance c) absence of stump pain d) amputation through or above the knee	12/13
Boutoille et al.(2008)^28^	MOS SF-36	Retrospective case control study	6 TTA (mean age 68 years) and 9 with a current foot ulcer, (mean age 70 years)	Evaluating the influence of amputation or conservative treatment for a diabetic foot ulcer on physical and social aspects of patients' QoL	TTA group reported less pain but similar QoL compared to foot ulcer patients	14/15
Fortington et al. (2013)^29^	RAND-36	Longitudinal study.	82 LLA. Mean age 67.8±13 years. 63% TTA and 37% TFA. A total of 35 remained in the study at 18 months follow up.	Evaluating how the age and walking distance could influence QoL 18 months after the amputation. Comparing QoL of LLA with a control group	Only the domain ±social function± was influenced significantly by the ability to walk. Except for physical function, the other domains were similar to population norm values. The domain ±physical function± was positively correlated to lower levels of amputation and to age categories of less than 65 years. QoL improved after amputation, in particular in the first 6 months	14/15
Knezevic et al. (2015)^30^	RAND-36	Cross-sectional study	28 LLA. 61% TFA, 39% TTA. Mean age: 65.4 ± 13.6 years.	Assessing the QoL of the patients with LLA compared to a control group, taking into account the influence of age and level of amputation	TTA are more mobile than TFA. The most significant difference was in the domains "physical functioning" and "general health", with higher scores reported by TTA	14/15

**Abbreviations**: LLA, lower limb amputees; TTA, transtibial amputees; RAND-36, Research and Development Corporation measure of Quality of Life 36-Item Health Survey 1.0; SF-36, Short-Form General Health Survey; MOS SF-36, Medical Outcomes Study 36-item short-form

**Table 3:** Overview of studies reporting satisfaction with the prosthesis data.

Authors	Evaluation tools	Study protocol	Characteristics of patients	Aim of study	Results	Critical Review Form-TOTAL items
Bonnet et al.(2015)^31^	Quebec User Evaluation of Satisfaction with Assistive Technology 2.0 questionnaire	Crossover study	12 dysvascular TTA, mean age 77 years	Evaluating the benefit of a NGF versus SACH foot for low-activity TTA	Higher satisfaction level using NGF compared to SACH. The increase is significant for the global score of the questionnaire.	14/15
Delussu et al. (2016)^32^	SATPRO	Observational study	20 TTA, mean age 66.6±6.7 years. 19 amputees had a K-level of 2 and 1 had a K-level of 1	Assessing amputees satisfaction with prosthesis using two different prosthetic feet: 1M10 Adjust and SACH in low-mobility TTA	Participants showed a significantly higher improvement in SAT-PRO with “1M10 Adjust” than with SACH.	12/13

**Abbreviations**: TTA: Trans-Tibial Amputee, SAT-PRO: Satisfaction with Prosthesis, SACH: Solid Ankle Cushion Heel, NGF: New Geriatric Foot.

### Patients Characteristics

Only three studies focused on TTA with a mean age>60 years, two of which primarily investigated the effects of a new prosthetic foot.^31,32^ Only one study exclusively assessed the QoL of a population of non-traumatic TTA who were successful prosthetic users.^2^ In most of the selected studies, the sample consisted in a mixed group of TTA and TFA. In two articles^25,27^ the sample also included partial foot amputees and in two other studies^28,30^ there was a control group consisting of people with intact lower extremities or foot ulcer.

### QoL and HS measurement

The most used tools were the Research and Development Corporation measure of Quality of Life 36-Item Health Survey 1.0 (RAND-36) (3 times) and the SF-36 (2 times).^34,35^ The RAND-36 is a self-reported questionnaire which includes the same items as those of SF-36, but scoring is slightly different in the domains “pain” and “general health”. PEQ and TAPES-R questionnaire was used one time (Table 4).

**Table 4:** Overview of the tools used for HS and QoL.

Evaluation tools	Authors
PEQ	Harness et al (2001)^2^
SWLS	Norvell et al (2011)^25^
WHO QOL-BREF	Cox et al (2011)^26^
TAPES-R	Quigley et al (2016)^27^
SF-36	Quigley et al (2016)^27^;Boutoille et al (2008)^28^; Knezevic et al (2015)^30^
RAND-36	Van der Schans et al (2002)^17^;Knezevic et al (2015)^30^; Fortington et al (2013)^29^

## DISCUSSION

The intention of this review was to evaluate the impact of trans-tibial amputation on the QoL, HS and prosthesis satisfaction, in order to determine the appropriate intervention to improve these aspects in older trans-tibial amputees. Many studies reported a better QoL and HS in TTA compared to TFA,^26^ particularly in the “physical functioning” domain.^29,30^ Moreover, TTA also had significantly higher scores for functional independence compared to the TFA.^26^ Considering that TTA have better QoL than TFA, we investigated whether these patients could maintain high QoL throughout the aging process.

Indeed, young age at the time of amputation was associated with better QoL in the categories of physical disability, energy level, emotional reactions and social isolation while advanced age was associated with reduced mobility and lower energy level than younger population.^6^ However, whether ageing affected QoL is still debated. A recent study stated that quality of life in LLA is significantly influenced by age,^36^ while Adegoke and co-workers (2012) reported that the patient’ age at the time of amputation did not affect general quality of life.^37^ In our review, we found that there are no longitudinal studies that describe changes in the quality of life during ageing. The “physical functioning” appears to be the main factor affecting QoL and satisfaction in older TTA. Indeed, Fortington et al., (2013) found that subjects over 65 years of age had lower outcome than younger amputees only for physical function, while other domains were comparable to population norm values.^29^

The walking distance aspect of mobility is one of the main factors to be considered when evaluating QoL after LLA.^17^ Elderly TTA with higher mobility scores were more likely to be satisfied with life,^25^ and perception of their social burden correlated strongly with their ability to walk using their prosthesis. Fortington et al.,^29^ reported that walking distance is associated with improved scores in social function. One study identified also that mobility capability was significantly influenced by these risk factors: age>65y, alcohol disorder, hypertension, anxiety or depression.^25^

Another aspect that was postulated to interfere with QoL was the level of pain. Rather controversially the results did not confirm this assumption. In fact, in the only study in which TTA alone were enrolled, Harness et al., (2001)^2^ found that the ability to walk using the prosthesis was correlated with the presence of pain and residual limb health.^2^ Moreover, the same study reported a correlation between the patient’s satisfaction and lesser pain level.^2^ Even Knežević and co-workers reported no differences between TTA and TFA on role limitations due to pain and physical health.^30^ On the contrary, another study described how the presence of phantom pain might imply a poorer health-related quality of life.^17^

An important role of pain was described by Boutoille et al.,(2008).^28^ The authors compared HS and pain in patients having experienced an amputation due to diabetic foot ulcer and patients suffering for a current foot ulcer with no previous history of amputation. They reported that a transtibial amputation allows similar HS with less pain with respect to a conservative, unsuccessful, treatment for diabetic ulcer.

Two studies focused on the effect of a prosthetic foot in hypomobile older TTA.^31,32^ Both studies investigated the performance and satisfaction utilizing different feet compared to the traditional SACH (solid ankle cushion heel) foot. The SACH is considered to be the most appropriate foot for hypomobile TTA and also the most prescribed foot as it is inexpensive, easy to use, and perceived as stable.^39^ Delussu et al.,(2016) tested the “1M10 Adjust” foot that is a multi-axial lightweight foot that allows stiffness heel adjustments to adapt to individual needs.^32^ In another study a new geriatric foot was evaluated which shape and type of foam in this foot allows to be shorter and lighter compared to SACH.^31^ Both studies reported greater patient satisfaction with the tested prosthetic feet. Moreover, the new geriatric foot reduced the mean pressure in the socket and the “1M10 Adjust” showed a statistically significant reduction of the energy cost of walking.^31,32^ The common feature between these two tested prosthetic feet is the lighter weight compared to SACH. This may lead us to hypothesize that lighter prosthetic components for hypomobile mature TTA could positively affect their satisfaction with the prosthesis.

In this review we selected only articles published from January 2000 to March 2019 which might be a limitation. We did not search studies prior to 2000 because of the important progress in technology of the socket and suspension system in the late 1990s (from Patellar Tendon Bearing to Total Surface Bearing).

## CONCLUSION

Our review has pointed out that there are very few studies that have investigated this particular population of amputees (TTA aged >60 years). Only one study exclusively investigated QoL in older TTA.^2^ We have observed authors have rarely used specific tools for measurement of QoL and HS in LLA. In general, the QoL and HS of LLA is influenced mostly by daily activities.^40^ Patients with amputation often encounter difficulties in everyday activities because they have lost their independence and must rely on others. This could influence negatively many aspects of their lives, such as social and financial. For this, it is very important, when studying QoL of amputees, to also analyse their social environment.^41^ On the basis of the main results of this review we can conclude that efforts have to be taken in order to improve mobility in TTA for a better QoL, by means of adequate rehabilitation, reduction of pain, and appropriate prosthetic components. The literature available on this specific population is insufficient and future research will benefit from more prospective observational cohort studies. Such studies will need to be conducted with appropriate inclusion criteria and larger sample sizes to better understand the QoL and HS in this population.

## DECLARATION OF CONFLICTING INTERESTS

The authors declared no potential conflicts of interest with respect to the research, authorship, and/or publication of this article.

## AUTHOR CONTRIBUTION

**Stefano Brunelli**: designed the study, online databases search and led the writing of the manuscript.

**Cinzia Bonanni**: online databases search, data extraction and supported the writing of the manuscript.

**Calogero Foti**: revised the manuscript critically for important intellectual content.

**Marco Traballesi**: revised the manuscript critically for important intellectual content.

## SOURCES OF SUPPORT

The authors received no financial support for the research, authorship, and/or publication of this article.

## ABBREVIATIONS

QoL: Quality of Life,
HS: Health Status,
LLA: Lower Limb Amputee,
TTA: Trans-Tibial Amputee,
TFA: Trans-Femoral Amputee,
TAPES: Trinity Amputation and Prosthesis Experience Scales,
PEQ: Prosthesis Evaluation Questionnaire,
WHO QOLBREF: World Health Organization Quality of Life Questionnaire,
SF-36: The 36-Item Short Form Health Survey,
NHP: Nottingham Health Profile,
RAND- 36: Research and Development Corporation measure of Quality of Life 36-Item Health Survey 1.0,
SAT-PRO: Satisfaction with Prosthesis,
NGF: New Geriatric Foot,
SWLS: Satisfaction with Life Scale.
